# A Rare Case of RYR2 Mutation Causing Sudden Cardiac Arrest Due to Catecholaminergic Polymorphic Ventricular Tachycardia

**DOI:** 10.7759/cureus.13417

**Published:** 2021-02-18

**Authors:** Lalitha Padmanabha Vemireddy, Ammar Aqeel, Grace W Ying, Delaram Majlesi, Vincent Woo

**Affiliations:** 1 Internal Medicine, Chicago Medical School Internal Medicine Residency Program at Northwestern McHenry Hospital, McHenry, USA; 2 Cardiology, Northwestern Medicine McHenry Hospital, McHenry, USA

**Keywords:** ryr-2 gene mutation, catecholaminergic polymorphic ventricular tachycardia

## Abstract

Catecholaminergic polymorphic ventricular tachycardia (CPVT) is a complex disorder that can induce lethal ventricular arrhythmias, secondary to activation of the sympathetic nervous system. This disease is often diagnosed in childhood but can also manifest in adulthood (the early 40s). Gene mutations such as CALM1, RYR2 (ryanodine receptor-2), CASQ2, and TRDN have been identified as common causes of CPVT. Those affected can present with episodes of syncope, sudden cardiac arrest, or sudden cardiac death due to either fast polymorphic ventricular tachycardia (VT) or bidirectional VT. Diagnosing and managing CPVT can often be challenging as patients are often asymptomatic and may present after a sudden cardiac arrest. Exercise stress testing and genetic testing play a pivotal role in the workup of CPVT. Avoidance of strenuous activities and pharmacological therapy with beta-blockers are the mainstays of treatment. Here, we report a case of CPVT in a patient with RYR2 gene mutation, causing sudden cardiac arrest.

## Introduction

Catecholaminergic polymorphic ventricular tachycardia (CPVT) is an inherited disorder characterized by electrical instability in the cardiac myocytes resulting from activation of the adrenergic system [1, 2]. CPVT has been noted to have an autosomal inheritance, with certain gene mutations being dominant, such as CALM1 and RYR2 (ryanodine receptor-2), while others have a recessive pattern, namely CASQ2 and TRDN [3]. CPVT is associated with syncope due to ventricular tachycardias, either fast polymorphic ventricular tachycardia (VT) or bidirectional VT [4]. The onset of symptoms for CPVT is predominantly seen early in life, ages seven to twelve; however, there are also reports of patients presenting as late as in the fourth decade of life [1]. In at least 30% of patients, a sudden cardiac arrest has been documented, while 80% of patients will experience at least one or more syncopal episodes [3]. This report presents a case of RYR2 gene mutation causing fast polymorphic VT resulting in sudden cardiac arrest at 46 years of age. 

## Case presentation

A 46-year-old female was brought to our hospital by an emergency medical technician after an out-of-hospital cardiac arrest. Field evaluation revealed an initial rhythm of ventricular fibrillation requiring unsynchronized defibrillation. While in the emergency department, the patient went into asystole, requiring multiple rounds of epinephrine and mechanical ventilation. Post-resuscitation, the patient was initiated on targeted temperature management (TTM). Initial electrocardiogram (EKG) after the return of spontaneous circulation (ROSC) showed sinus bradycardia with a QTc of 609 msec (normal QTc at baseline).

**Figure 1:**
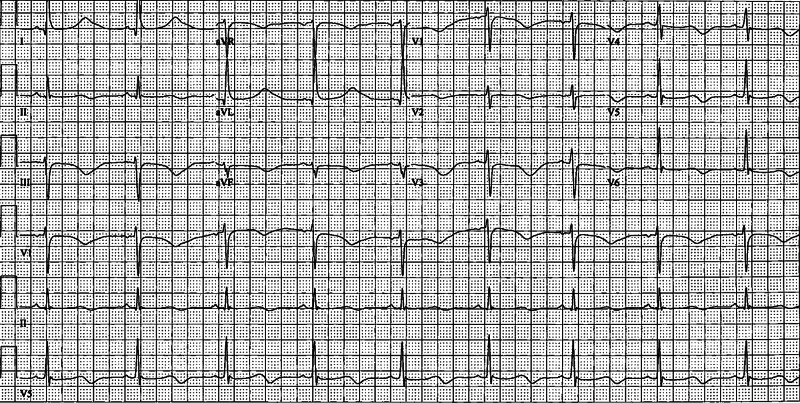
Initial EKG post-ROSC showing sinus bradycardia with a measured QTc of 609 msec EKG - electrocardiogram; ROSC - return of spontaneous circulation

Vitals post-ROSC included a temperature of 95.7 F, heart rate of 56 bpm, blood pressure of 97/69 mmHg, respiratory rate of 10/min, and oxygen saturation of 96% via Ambu bag. Initial workup, including but not limited to complete blood count, comprehensive metabolic panel, and other electrolytes, troponins, brain natriuretic peptide (BNP), were within normal limits. A transthoracic echocardiogram (TTE) revealed a decreased ejection fraction (EF) of 45% and a hypokinetic infero-septal wall. Post-TTM cardiac catheterization was pursued, revealing normal coronary anatomy without evidence of thrombosis. After the patient was extubated, she had multiple runs of VT, prompting an electrophysiologic (EP) study. The study was conducted with ventricular stimulation protocol, which showed easily inducible fast VT, abruptly changing into multiple morphologies.

**Figure 2:**
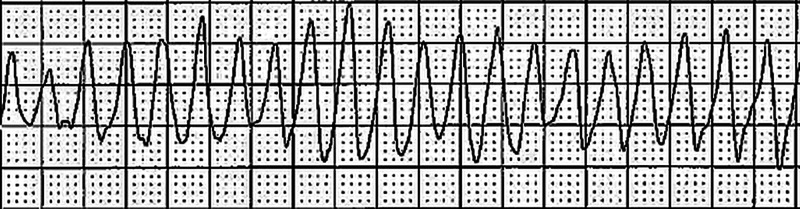
Rhythm shown is polymorphic ventricular tachycardia (PMVT) reproduced during the electrophysiological study

Subsequently, a single chamber automatic implantable cardioverter-defibrillator (AICD) was placed, and the patient was discharged on metoprolol tartrate 25 mg twice daily. Repeat TTE was done after AICD placement, which demonstrated an EF of 60% with no regional wall motion abnormalities. Comprehensive genetic testing was done in the outpatient setting, which revealed that the patient and her father were positive for RYR2 gene deletion. After about two years on maintenance beta-blockade therapy, she was noted to have a syncopal episode, which necessitated inpatient admission and AICD interrogation. AICD interrogation revealed that the patient had multiple episodes of PMVT, which was also noted to degenerate into an episode of ventricular fibrillation (VFib), requiring an AICD shock. During this hospitalization, the patient continued to have episodes of PMVT, the decision was made to transition metoprolol to nadolol 80 mg daily, and verapamil 120 mg daily was also added. After these adjustments were made to her medication regimen, the patient did not have any further hospital admissions.

## Discussion

CPVT is a rare inherited disorder that affects one out of every 10,000 people. It is characterized by an excessive sympathetic tone (emotional or physical) leading to lethal ventricular arrhythmias [1]. As described before, CPVT is inherited in an autosomal fashion, with gene mutations in CALM1 and RYR2 being dominant and CASQ2 and TRDN being recessive [3]. Out of all documented CPVT cases, the RYR2 mutations encompass 60% of cases [5]. Activation of ryanodine (RYR2) receptors secondary to L-gated calcium (Ca) channel activation leads to Ca influx into myocytes resulting in electrical and muscular contractility of the heart [6]. Mutations in the RYR2 gene result in prolonged calcium channel opening with subsequent calcium leak during diastole leading to an increased risk of ventricular arrhythmias and death [5]. Major symptomatology includes syncope, sudden cardiac arrest and death, while minor symptoms include dizziness, palpitations, and chest pain. Common arrhythmias include PMVT or bidirectional VT, both of which can propagate into VFib [4]. Electrocardiographic features of bidirectional VT include reversal or alternation of QRS complexes close to 180 degrees or alternating bundle branch blocks [7]. Diagnosing CPVT with standard ECG can be challenging as most of these patients usually do not have any baseline abnormalities at rest [2]. Sinus bradycardia with a presence of U waves can be seen often in patients with CPVT [8]. Exercise stress testing can be an important diagnostic test for CPVT as ventricular arrhythmias are manifested with an increased heart rate (110-130 beats per minute). The arrhythmias can gradually resolve as the heart rate slows down. An important consideration is that negative stress testing does not completely rule out CPVT. Extended cardiac monitoring devices can be useful in instances where stress testing is inconclusive [2].

Diagnostic criteria proposed by Prior et al. include [9]:

1) the presence of a normal structural heart and normal EKG and unexplained stress-induced bidirectional VT or PMVT in individuals younger than 40 years old;

2) finding a pathogenic mutation;

3) and the presence of unexplained stress-induced bidirectional VT or PMVT in a first-degree family member, who has no structural heart abnormalities;

4) presence of a normal structural heart and normal EKG and unexplained stress-induced bidirectional VT or PMVT in individuals older than 40 years old.

Patients diagnosed with CPVT are highly recommended to avoid physical stress, such as participating in high-intensity exercise and any form of activities involving high adrenergic output. It is also imperative that patients are counseled on strict medication adherence [1, 4]. Pharmacologically, beta-blockers have been the mainstay of the treatment of CPVT. Of these medications, the long-acting beta-blocker nadolol is preferred [10]. Hayashi et al. conducted an observational study, which revealed that patients taking beta-blockers had fewer arrhythmic events than patients without treatment (58% in eight years in the untreated group vs. 27% in the treated group, p=0.001). Nadolol has been proven to be superior to other beta-blockers to prevent arrhythmic events (19% while on nadolol, vs. 39% with other beta-blocking medications) [11]. One of the main reasons why nadolol seems to be superior in treating such arrhythmias is because of once-a-day dosing, leading to improved compliance compared to other beta-blockers. Carvedilol has also been shown to be effective by inhibiting RYR2 activity [4]. Almost 30-40% of patients experience symptoms despite beta-blockers, requiring implantable cardioverter-defibrillator (ICD) therapy [1]. Calcium channel blockers like verapamil have also been used to treat ventricular arrhythmias in CPVT, especially in patients with CASQ2 gene mutation [1, 2, 4]. Flecainide has been used in patients with CPVT when arrhythmias are refractory to treatment with first or second-line medical therapy. It has also been shown that flecainide and propafenone directly inhibit RYR2 channels and prevent arrhythmias. Inhibition of the sodium-calcium exchanger can indirectly decrease calcium by reducing sodium and RYR2-calcium antagonism, which is another possible mechanism of action of flecainide [2, 4]. Kannankeril et al. conducted a randomized controlled trial in patients with CPVT and demonstrated that flecainide, in combination with beta-blockers, showed a decrease in ventricular ectopic beats in comparison to the placebo with the beta-blocker group or the beta-blocker group alone [12]. Sympathetic denervation involves resection of the lower half of the stellate ganglion and T2-T4 sympathetic ganglion. Despite the lack of evidence regarding safety or efficacy, sympathetic denervation has been useful in patients with life-threatening uncontrolled arrhythmias on maximal medical therapy. Lastly, ICD can be considered in patients if symptoms persist despite optimal medical therapy [2, 4]. Genetic counseling is essential for first-degree relatives, as they should be evaluated for mutations to decrease the risk of future unwarranted and preventable adverse events [13].

## Conclusions

CPVT is a complex disease that carries a risk of sudden cardiac death in various age groups, with those at risk, including the younger population. For this reason, these patients must be screened and diagnosed promptly. A detailed and thorough history encompassing the patient's family can help lead the clinician to a proper workup to help diagnose CPVT. Exercise stress testing is often used to diagnose CPVT, and at times may be inconclusive, which leads to further electrophysiologic testing. Genetic testing can be useful in identifying the mutation causing CPVT. RYR2 gene mutation is a significant cause of CPVT, which has gained traction as one of the most difficult to diagnose, especially because most of these patients are asymptomatic. Beta-blockers, calcium channel blockers and, flecainide are the main pharmacological agents employed, with sympathetic denervation and ICD being used in refractory cases.
